# The validation, reliability, and measurement invariance of the relationship sabotage scale in Chinese college students

**DOI:** 10.1186/s40359-026-04546-x

**Published:** 2026-04-24

**Authors:** Wei Feng, Manyu Zhang, Changle Wang, Yubo Bu

**Affiliations:** 1 Jiangsu Food and Pharmaceutical Science College, Huai’an City, Jiangsu Province 223003 China; 2 Taizhou University, Taizhou City, Jiangsu Province 225300 China; 3 Wuhan University, Wuhan City, Hubei Province 430072 China

**Keywords:** Relationship sabotage, Psychometric properties, Measurement invariance, Chinese college students

## Abstract

**Background:**

Relationship self-sabotage has been primarily examined in Western contexts, and its measurement in Chinese populations remains limited. This study aimed to evaluate the psychometric properties of the Relationship Sabotage Scale (RSS) among Chinese college students.

**Methods:**

Two independent samples of Chinese college students with prior romantic experience were recruited from four universities in Jiangsu Province in 2024 (Sample 1: *n* = 550; Sample 2: *n* = 568). Confirmatory factor analysis was conducted to examine the factor structure. Measurement invariance was tested across gender, only-child status, migration experience, left-behind experience, and family residence. Reliability and multiple forms of validity, including convergent, discriminant, and known-groups validity, were assessed.

**Results:**

A three-factor structure—Defensiveness, Trust Difficulty, and Lack of Relationship Skills—was generally supported, with acceptable model fit overall (*χ*^*2*^*/df* = 3.81, RMSEA = 0.070, CFI = 0.951, TLI = 0.930), although model fit in Sample 1 was marginal. Measurement invariance was supported at the configural, metric, and scalar levels across the examined groups. The scale demonstrated acceptable internal consistency (α = 0.79–0.95), although corrected item–total correlations indicated variability across items, consistent with the multidimensional structure. Convergent validity was supported by positive associations with attachment avoidance and anxiety, whereas discriminant validity was indicated by negative associations with relationship satisfaction and self-esteem. Known-groups validity was partially supported, with some group differences observed across gender and family residence.

**Conclusions:**

These findings provide preliminary evidence supporting the reliability and validity of the RSS in a Chinese context. However, given the marginal model fit in one sample and variability in item-level performance, further validation in more diverse and representative samples is warranted. The scale may serve as a useful tool for assessing self-sabotaging tendencies in romantic relationships among Chinese college students.

**Supplementary Information:**

The online version contains supplementary material available at 10.1186/s40359-026-04546-x.

## Introduction

Intimate relationships represent not only a journey of mutual companionship and shared growth but also embody the profound significance of interdependence between partners [12, 13]. During the development of these relationships, partners, through their shared life experiences, engage in communication, demonstrate mutual tolerance, and foster trust and commitment across cognitive, emotional, and behavioral domains [4, 20, 52]. The maintenance and development of intimate relationships are closely associated with partners’ experiences of intimacy, relationship satisfaction [33], the mental health of both partners [6], and attachment styles formed in childhood [49]. Relationships characterized by positive experiences and high satisfaction are less susceptible to conflict and more likely to endure over time [21]. As a critical factor in sustaining intimate relationships [3], a persistent decline in relationship satisfaction often foreshadows the dissolution of the relationship.

Individuals’ attachment styles [47], attachment motivations, conflict strategies, self-disclosure [19], childhood experiences [17], and self-sabotaging behaviors in intimate relationships [38] can influence relationship satisfaction. These attachment-related processes are also closely linked to self-sabotaging tendencies in intimate relationships. Attachment Theory suggests that early childhood relationships with caregivers form a critical foundation for establishing intimate relationships in adulthood [7]. Bowlby [8] classified attachment types into secure and insecure, noting that early insecure attachment can impact individuals throughout their lives [14]. A cross-sectional study of students aged 18–28 revealed that individuals with insecure attachment patterns (anxious and avoidant) face challenges in maintaining long-term, stable intimate relationships [45].

Self-sabotage, as a prevalent cognitive strategy, exerts a continuous influence on intimate relationships [39]. Since Berglas and Jones [5] introduced the concept of self-handicapping, research has primarily focused on education, sports, and organizational contexts [48], with limited exploration of self-sabotage in intimate relationships. Although relationship self-sabotage has been conceptually defined and measured using the Relationship Sabotage Scale (RSS), existing research has primarily focused on Western and a limited number of non-Western cultural contexts. Thus, it remains unclear whether the construct and its measurement demonstrate comparable psychometric properties in Chinese or broader East Asian populations. Post (1988) first applied self-handicapping to interpersonal relationships, explaining behaviors during conflict resolution, where individuals may engage in subconscious, chronic self-handicapping for self-protection [31]. Such behaviors negatively impact relationship establishment and maintenance, often leading to relationship dissolution [40]. Grounded in Attachment Theory and Goal Orientation Theory, Peel et al. [38] defined relationship self-sabotage as “cognitive, emotional, and behavioral patterns that repeatedly undermine the establishment or maintenance of intimate relationships due to self-protective motives”. Such behaviors can cause emotional distancing and trust deficits between partners [31], contributing to relationship breakdown and potentially trapping individuals in a vicious cycle of relationship destruction, hindering the formation of stable, healthy intimate relationships [38].

To systematically assess the extent of self-sabotage in intimate relationships and identify relevant attitudes and behaviors for clinical interventions, Peel et al. [38] conducted interviews with Australian psychologists, identifying three core dimensions of relationship sabotage: Defensiveness, Trust Difficulty, and Lack of Relationship Skills. Through exploratory factor analysis (EFA) and confirmatory factor analysis (CFA), they developed the 12-item Relationship Sabotage Scale (RSS), which demonstrated good model fit (*GFI* = 0.96, *CFI* = 0.96, *TLI* = 0.95) and robust internal consistency and construct validity [40]. While the RSS has already shown strong psychometric properties in English-speaking populations and has been validated in cultural contexts such as Turkey and Iran, retaining its original items and three-factor structure [32, 44], its applicability in East Asian cultures, particularly among Chinese populations, has not yet been empirically established. Intimate relationships in China are shaped by collectivism [53], family and kinship ties, and cultural concepts such as renqing (interpersonal favor) and mianzi (face), which may result in unique manifestations of self-sabotaging behaviors. For example, cultural norms emphasizing relational harmony and face maintenance may particularly influence expressions of defensiveness and trust-related concerns [50], potentially altering the functioning or salience of these dimensions in Chinese contexts. Specifically, in a collectivistic cultural context that emphasizes relational harmony and face maintenance, individuals may be more likely to suppress direct confrontation [25, 50], which could result in more implicit or indirect forms of defensiveness rather than overt expressions. Similarly, trust-related concerns may be shaped by broader relational networks and social expectations, potentially manifesting differently compared to Western individualistic contexts [2]. In contrast, deficiencies in relationship skills may be more strongly influenced by socially learned norms and family-based interaction patterns [54]. These considerations suggest that the relative salience and expression of the RSS dimensions may vary across cultural contexts, underscoring the need for empirical validation. Moreover, relationship sabotage may overlap with other psychological symptoms, highlighting the importance of accurate assessment tools in Chinese populations [38].

This study focuses on Chinese university students with prior romantic relationship experience. This population was chosen because they are at a critical developmental stage for intimate relationships, and their behaviors may be influenced by both traditional collectivistic norms and contemporary social changes. Specifically, the study aims to:(1)Adapt the Chinese version of the RSS to ensure semantic equivalence and cultural appropriateness;(2)Validate the psychometric properties of the Chinese RSS, including its dimensional structure, reliability, and construct validity;(3)Test the measurement invariance of the Chinese RSS across key demographic variables, including gender, left-behind experiences, migration experiences, long-term family residence, and only-child status. For instance, gender differences have been consistently associated with variations in emotional expression, attachment-related responses, and conflict management strategies in intimate relationships [15, 30], which are directly relevant to self-sabotaging behaviors. Migration and left-behind experiences, particularly in the Chinese context, have been linked to disruptions in early attachment relationships and long-term effects on interpersonal trust and emotional regulation [10, 56], which may increase vulnerability to maladaptive relational patterns. In addition, only-child status may shape family interaction styles [29], dependence on parental support, and expectations in close relationships, while long-term family residence (urban vs. rural) reflects broader differences in socialization environments, access to resources, and relational norms [55]. Collectively, these factors are theoretically and empirically associated with individual differences in interpersonal functioning and are therefore relevant for examining measurement invariance of relationship self-sabotage across groups. Based on these considerations, we hypothesized that the Chinese RSS would retain its original three-factor structure and demonstrate satisfactory reliability and validity, and that measurement invariance would be supported across the examined demographic groups.

By addressing these aims, this research provides preliminary evidence regarding the cross-cultural applicability of the RSS and offers a potential tool for assessing relationship self-sabotage in Chinese university students. It also supports future cross-cultural comparisons and research on intimate relationships.

## Method

### Participants

**Fig. 1:**
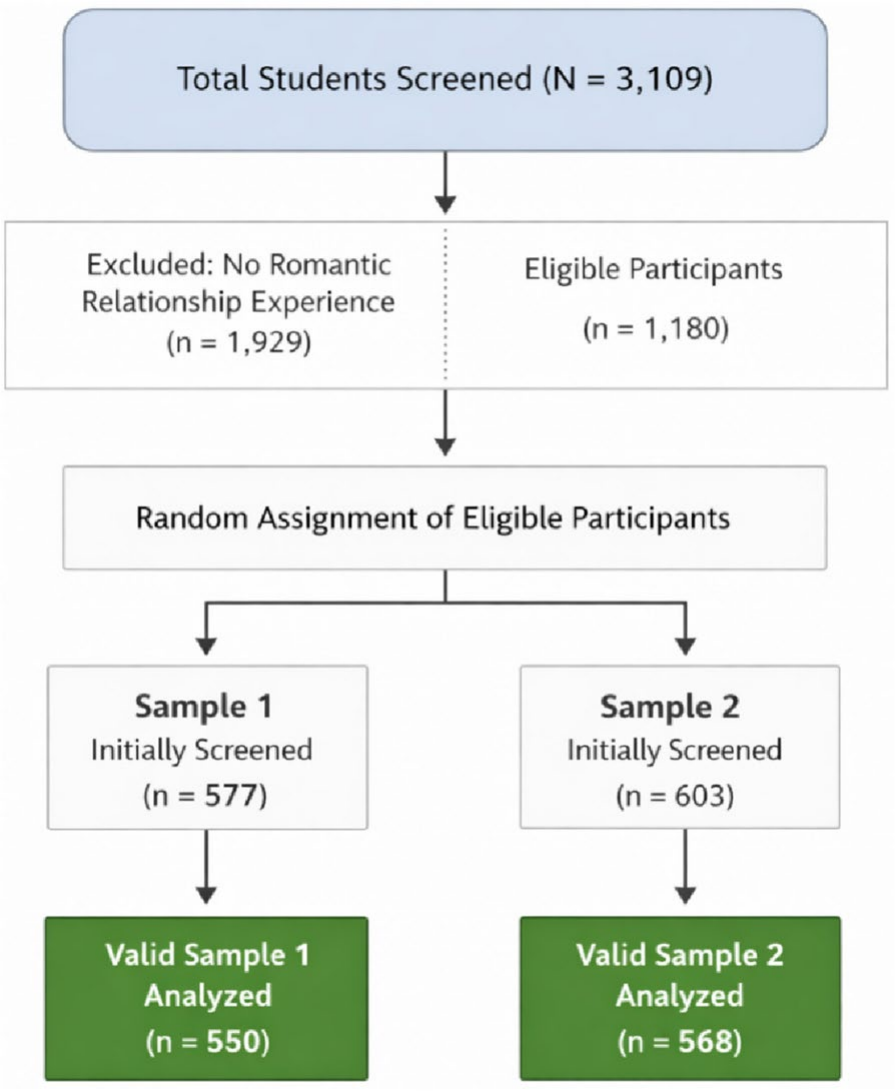
Flow diagram of participant selection and sample construction

Participants were drawn from a larger sample of 3,109 undergraduate students recruited from four universities in Jiangsu Province, China. Only students who reported having prior romantic relationship experience were included in the present study. The final analytic sample consisted of 1,118 participants after excluding invalid responses(selection process is presented in Fig. 1).

The sample was divided into two independent subsamples. Sample 1 included 550 participants (240 males, 310 females; *M*_age_ = 19.18 years, *SD* = 1.65). Sample 2 included 568 participants (240 males, 328 females; *M*_age_ = 18.82 years, *SD* = 1.51).

In Sample 1, 105 participants were only children, 309 were from rural areas, 151 reported left-behind experiences, and 96 reported migration experiences. In Sample 2, 111 participants were only children, 252 were from rural areas, 168 reported left-behind experiences, and 130 reported migration experiences (Table 1). These grouping variables were selected based on theoretical and empirical considerations described in the Introduction and were used to test measurement invariance of the RSS across demographic subgroups.

**Table 1:** Characterization of the samples

	Sample 1(*n* = 550)	Sample 2(*n* = 568)
	Frequency(% of the sample)	Frequency(% of the sample)
Age:	*M* = 19.18(*SD* = 1.65)	*M* = 18.82(*SD* = 1.51)
Gender:		
Male	240(43.64)	240(42.25)
Female	310(56.36)	328(57.75)
Only child:		
Yes	105(19.09)	111(19.54)
No	445(80.91)	457(80.46)
Migration experience:		
Yes	096(17.45)	130(22.89)
No	454(82.55)	438(77.11)
Left-behind experience:		
Yes	151(27.45)	168(29.58)
No	399(72.55)	400(70.42)
long-term family residence:		
Urban	241(43.82)	316(55.63)
Rural	309(56.18)	252(44.37)

Sample 1 was used for exploratory and structural validity analyses. Sample 2 was used to examine structural validity, convergent validity, discriminant validity, known-groups validity, and reliability.

### Measures

#### Relationship sabotage scale (RSS)

The Chinese version of the Relationship Sabotage Scale (RSS), originally developed by Peel et al. [38], was adapted with authorization. The translation and cultural adaptation followed a multi-step procedure consistent with established cross-cultural instrument adaptation guidelines. First, three psychology faculty members and six graduate students independently translated the original English items into Chinese. Discrepancies were resolved through structured discussion to produce a preliminary version. Second, two psychology faculty members with overseas academic experience independently conducted back-translation. Semantic inconsistencies between the back-translated and original versions were reviewed and reconciled. Third, content validity evaluation was conducted by an expert panel consisting of one associate professor in clinical and counseling psychology and four faculty members specializing in psychological measurement and relationship research. Experts independently rated each item for conceptual relevance to its intended dimension using a 4-point relevance scale (1 = not relevant, 4 = highly relevant). The item-level content validity index (I-CVI) was calculated, with 0.78 adopted as the minimum acceptable threshold. Item classification required convergence across three criteria:(a) I-CVI ≥ 0.78; (b) expert agreement rate ≥ 80%; (c) theoretical coherence within the Chinese cultural context.

During this process, Item 5 showed stronger conceptual alignment with the Defensiveness dimension than with Trust Difficulty. More than 80% of experts classified the behavioral content of this item as reflecting defensive relational strategies rather than mistrust per se. Accordingly, the item was reassigned prior to statistical validation. Finally, 12 psychology master’s students completed the revised Chinese RSS, followed by cognitive interviews to assess clarity, wording, and response format. Minor linguistic refinements were made based on feedback, resulting in the finalized Chinese RSS. The Chinese RSS comprises 12 items across three dimensions: Defensiveness, Trust Difficulty, and Lack of Relationship Skills. It uses a 7-point Likert scale (1 = strongly disagree, 7 = strongly agree), with Items 9, 10, 11, and 12 reverse-scored. Higher scores indicate more pronounced self-sabotaging behaviors in intimate relationships.

#### Experiences in close relationships inventory (ECR)

The Chinese version of the Experiences in Close Relationships Inventory [28] was used to measure attachment avoidance and attachment anxiety. The ECR includes 36 items across two dimensions (odd-numbered items for attachment avoidance, even-numbered items for attachment anxiety), using a 7-point Likert scale (1 = strongly disagree, 7 = strongly agree). Items 3, 15, 19, 22, 25, 27, 29, 31, 33, and 35 are reverse-scored. Higher scores indicate greater levels of attachment avoidance or anxiety. In this study, the ECR demonstrated a Cronbach’s *α* of 0.858.

#### Relationship satisfaction scale

The Chinese version of the Relationship Satisfaction Scale [46] was used to assess satisfaction in intimate relationships. This 7-item scale (e.g., “Our relationship will continue to develop well,” “I don’t receive the love I deserve from my partner”) uses a 5-point Likert scale (1 = strongly disagree, 5 = strongly agree), with Items 5, 6, and 7 reverse-scored. Higher scores indicate greater relationship satisfaction. In this study, the scale’s Cronbach’s *α* was 0.842.

#### Self-esteem scale

The Self-Esteem Scale [43] was used to measure self-esteem. This 10-item scale (e.g., “I feel that I am a person of worth, at least on an equal plane with others,” “I am able to do things as well as most other people”) uses a 4-point Likert scale (1 = strongly disagree, 4 = strongly agree), with Items 3, 5, 8, 9, and 10 reverse-scored. Higher scores indicate higher self-esteem. The Chinese version revised by Wei, Zhang, and Mao [51] was used, which has been validated in Chinese adolescent populations. In Peng et al. [41], the scale demonstrated good reliability. In this study, the scale’s Cronbach’s *α* was 0.838.

### Procedure

From January to February 2024, the research team obtained authorization from the scale developers to revise the Chinese version of the RSS. Ethical approval was granted by the university’s Academic Ethics Committee. With permission from local education authorities, cluster sampling was employed. We randomly selected 20 classes from each of four universities located in Huai’an, Taizhou, and Suzhou, Jiangsu Province (representing northern, central, and southern Jiangsu, respectively, with increasing levels of economic development from north to south). Five classes were randomly selected from each grade level, and each class included between 28 and 47 students. In total, 3,109 questionnaires were collected.

Data were collected on-site in classroom settings. Participants accessed an anonymous electronic questionnaire by scanning a QR code provided by the research team and completed the survey independently using their mobile devices. The questionnaire required approximately 15–20 min to complete under classroom supervision.

Informed consent was obtained prior to participation, and students were informed of their right to withdraw at any time without penalty. All responses were anonymous and confidential. The last four digits of participants’ phone numbers were collected solely for research matching purposes and were stored separately from survey responses. To ensure data quality, screening items were included to identify and exclude invalid responses. In addition, only participants who indicated that they had prior romantic experience were eligible for inclusion in the study.

As compensation, participants could choose one of the following rewards: educational materials or instructional videos, a music bundle, or a psychology-related book. Participants who withdrew before completing the survey were still provided with an English learning video.

### Data analysis

Data from Samples 1 and 2 were analyzed using SPSS 27.0 and Mplus 8.3. Preliminary analyses included descriptive statistics and item analysis. Item discrimination was examined using corrected item–total correlations, computed by removing each item from its respective subscale score to avoid part–whole inflation.

Structural validity was evaluated via confirmatory factor analysis (CFA) in Mplus 8.3 using robust maximum likelihood estimation (*MLR*). Model fit was evaluated using multiple indices, including the Comparative Fit Index (*CFI*), the Tucker–Lewis Index (*TLI*), the Root Mean Square Error of Approximation (*RMSEA*), and the Standardized Root Mean Square Residual (*SRMR*). Following Hu and Bentler [22] and Jebb et al. [23]), values of *CFI* and *TLI* ≥ 0.90 (≥ 0.95 indicating good fit), *RMSEA* ≤ 0.08 (≤ 0.06 indicating good fit), and *SRMR* ≤ 0.08 were considered indicative of acceptable model fit. Model fit was interpreted in light of sample size, model complexity, and the presence of marginal fit in Sample 1, consistent with recent methodological recommendations (Marsh et al., [34];McNeish, [35]).

In addition to the hypothesized three-factor model, alternative modeling approaches, were explored to examine whether a more complex structure would provide a better fit to the data. However, these methods encountered convergence difficulties, non-estimable standard errors, and unstable factor loadings in our data. Consistent with previous studies (Morin, Myers, & Lee, [37]), these results indicate that overly complex Bi-factor models may not be suitable under certain data conditions. Therefore, we retained the original CFA model as the primary analytic framework. Details on model attempts and rationale for selecting the original CFA model are provided to ensure transparency and interpretability. Reliability was evaluated using both Cronbach’s *α* and McDonald’s *ω* coefficients to provide robust estimates of internal consistency.

Supplementary evidence of known-groups validity was examined through correlations with attachment avoidance, attachment anxiety, relationship satisfaction, and self-esteem. In addition, independent-samples t-tests were conducted to assess group differences in gender, long-term family residence, only-child status, left-behind experiences, and migration experiences. These analyses provide supplementary external validity evidence, complementing the structural validity established via CFA.

The RSS was tested for measurement invariance across five demographic variables: gender, long-term family residence, only-child status, left-behind experiences, and migration experiences. These variables were selected based on prior research suggesting that demographic characteristics, family background, and childhood experiences can influence self-sabotaging behaviors in romantic relationships. Testing invariance across these groups allows us to examine whether the scale measures relationship sabotage consistently across participants with different social and familial experiences. Measurement invariance of the RSS across gender, long-term family residence, only-child status, left-behind experiences, and migration experiences was examined using multi-group confirmatory factor analysis (CFA) within a structural equation modeling framework. Invariance was tested sequentially at three levels: (1)Configural invariance, which assesses whether the factor structure (i.e., number of factors and item-factor loadings) is consistent across groups; (2)Metric invariance, which constrains factor loadings to be equal across groups, testing whether the items are interpreted similarly; and (3)Scalar invariance, which further constrains item intercepts, allowing for valid comparison of latent means across groups.

Model fit changes between successive models were evaluated using *ΔCFI*, *ΔRMSEA*, and *ΔSRMR*, following Chen & F.F [9] and Cheung & Rensvold [11]. Thresholds for invariance were set as follows: for configural → metric, *ΔCFI* ≤ 0.010, *ΔRMSEA* ≤ 0.015, and *ΔSRMR* ≤ 0.030; for metric → scalar, *ΔCFI* ≤ 0.010, *ΔRMSEA* ≤ 0.015, and *ΔSRMR* ≤ 0.010. These procedures ensure a rigorous, transparent assessment of measurement invariance across demographic groups.

### Ethics approval

Our study received ethical approval from the Academic Ethics Committee of Huaiyin Normal University on January 5, 2024 (Approval No. HNU202401051136). All procedures followed the ethical standards of the university, national research guidelines, and the principles of the 1964 Helsinki Declaration and its later amendments.

All participants in this study were adults according to Chinese law, meaning they were 18 years of age or older. No minors took part in the research. Before completing the survey, every participant was informed of the purpose of the study, their rights, and the voluntary nature of participation. All participants provided written informed consent and signed the consent form.

## Results

### Item analysis

Initial analyses examined the correlations between each item and its corresponding subscale score. The uncorrected item–total correlations ranged from 0.766 to 0.952 (Table 2). These values were likely inflated due to part–whole overlap, as each item was included in the subscale total. To address this issue, corrected item–total correlations were recalculated by excluding each item from its corresponding subscale score. The corrected coefficients are reported in Table 3 and provide a more accurate estimate of item homogeneity.

**Table 2:** Uncorrected Item–Total Correlations for the RSS (for reference only)(*n* = 568)

Defensiveness		Trust Difficulty		Lack of Relationship Skills	
Item	*r*	Item	*r*	Item	*r*
Q1	0.897^***^	Q5	0.766^***^	Q9	0.909^***^
Q2	0.931^***^	Q6	0.864^***^	Q10	0.952^***^
Q3	0.938^***^	Q7	0.843^***^	Q11	0.936^***^
Q4	0.928^***^	Q8	0.786^***^	Q12	0.927^***^

^***^P < 0.001, These correlations are inflated due to part–whole overlap and are reported for descriptive purposes only

**Table 3:** Item Analysis and Internal Consistency for Each Subscale of the RSS(*n* = 568)

Item	Scale Mean if Item Deleted	Scale Variance if Item Deleted	Corrected Item-Total Correlation	Cronbach's *α* if Item Deleted
Q1	43.48	93.84	0.52	0.71
Q2	44.17	98.29	0.47	0.72
Q3	44.15	99.44	0.39	0.73
Q4	43.63	92.22	0.53	0.70
Q5	44.68	108.60	0.20	0.74
Q6	43.95	95.84	0.47	0.71
Q7	42.31	89.53	0.56	0.70
Q8	42.30	91.15	0.55	0.70
Q9	40.96	104.26	0.27	0.74
Q10	40.69	109.99	0.14	0.75
Q11	40.78	108.51	0.16	0.75
Q12	40.71	110.23	0.14	0.75

As shown in Table 3, most items (Q1–Q4, Q6–Q8) exhibited corrected item–total correlations ranging from 0.39 to 0.56, indicating acceptable item homogeneity. Several items (Q5, Q9–Q12) had lower correlations (0.14–0.27), which may reflect the multidimensional structure of the scale, as the total score aggregates across distinct subdimensions. Deletion of any single item had minimal effect on Cronbach’s α (0.70–0.75), confirming that no item substantially compromises the overall reliability of the scale.

Taken together, these findings suggest that all items can be retained. The lower correlations of some items reflect the multidimensional structure of the scale rather than calculation artifacts. By presenting both Tables 2 and 3, we ensure transparency and address reviewers’ concerns regarding inflated correlations.

### Validity analysis

CFA with robust maximum likelihood estimation was conducted on Sample 1 (*n* = 550) based on the original RSS structure (Items 1–4: Defensiveness; Items 5–8: Trust Difficulty; Items 9–12: Lack of Relationship Skills). Results showed factor loadings ranging from 0.549 to 0.953, with *χ*^*2*^ = 421.832,* χ*^*2*^*/df* = 8.27, *RMSEA* = 0.115 (90% CI: 0.105–0.125), *CFI* = 0.842, and *TLI* = 0.796, indicating poor model fit.

Item 5 (“I often feel jealous of my partner”) was reassigned from Trust Difficulty to Defensiveness based on expert review and cross-cultural considerations. In the Chinese context, expressions of jealousy are more closely related to self-protective or defensive behaviors, rather than trust-related concerns. CFA results were consistent with this reassignment, as Item 5 showed higher factor loadings on Defensiveness than on Trust Difficulty.

The RSS structure was revised accordingly (Items 1–5: Defensiveness; Items 6–8: Trust Difficulty; Items 9–12: Lack of Relationship Skills), and CFA was repeated on Sample 1. Results showed factor loadings of 0.654–0.951, *χ*^*2*^ = 287.77, *χ*^*2*^*/df* = 5.64, *RMSEA* = 0.092 (90% CI: 0.082–0.102), *SRMR* = 0.108, *CFI* = 0.899, and *TLI* = 0.870, indicating marginal acceptable model fit.

CFA on the revised structure was then conducted on Sample 2 (*n* = 568), yielding factor loadings of 0.763–0.948, *χ*^*2*^ = 247.34, *χ*^*2*^*/df* = 4.85, *RMSEA* = 0.082 (90% CI: 0.072–0.093), *SRMR* = 0.084, *CFI* = 0.925, and *TLI* = 0.904, indicating acceptable overall model fit. Alternative models (Bi-factor CFA and ESEM) were tested but failed to converge or produced unstable estimates; thus, the revised CFA model was retained.

Pearson correlations revealed significant relationships between RSS dimensions: Defensiveness was positively correlated with Trust Difficulty (*r* = 0.620, *P* < 0.001) and Lack of Relationship Skills (*r* = 0.577, *P* < 0.001), while Trust Difficulty was negatively correlated with Lack of Relationship Skills (*r* = −0.431, *P* < 0.001).

### Convergent and discriminant validity

The Experiences in Close Relationships Inventory (attachment avoidance and anxiety) was used as a criterion for convergent validity, while the Relationship Satisfaction Scale and Self-Esteem Scale were used for discriminant validity. As shown in Table 4, the RSS dimensions (Defensiveness, Trust Difficulty, Lack of Relationship Skills) were significantly correlated with attachment avoidance and anxiety (absolute *r* values: 0.097–0.716). Of the six correlations with relationship satisfaction and self-esteem, five were significant, with only the correlation between Trust Difficulty and relationship satisfaction being non-significant.

**Table 4:** Convergent and Discriminant Validity of the Relationship Sabotage Scale(*n* = 568)

Variable	Attachment Avoidance	Attachment Anxiety	Relationship Satisfaction	Self-Esteem
Relationship Sabotage	0.402^***^	0.518^***^	−0.458^***^	−0.541^***^
Defensiveness	0.250^***^	0.662^***^	−0.296^***^	−0.463^***^
Trust Difficulty	−0.097^*^	0.716^***^	0.055	−0.244^***^
Lack of Relationship Skills	0.494^***^	−0.418^***^	−0.507^***^	−0.226^***^

^*^P < 0.05, ^**^P < 0.01, ^***^P < 0.001

### Known-groups validity evidence

Independent-samples t-tests revealed significant gender differences in Relationship Sabotage, Defensiveness, and Trust Difficulty, but not in Lack of Relationship Skills (Table 5). Lack of Relationship Skills showed significant differences by long-term family residence (Table 6). No significant differences were observed for only-child status, migration experiences, or left-behind experiences (Tables 7, 8 and 9). These findings are generally consistent with theoretical expectations and provide supplementary evidence supporting the scale’s known-groups validity.

**Table 5:** Gender Differences in Relationship Sabotage(*n* = 568)

Variable	Male(*n* = 240)	Female(*n* = 328)	*t*	*P*
Relationship Sabotage	3.51 ± 0.92	3.18 ± 0.89	4.31	< *0.001*
Defensiveness	3.57 ± 1.66	2.93 ± 1.36	5.10	< *0.001*
Trust Difficulty	4.12 ± 1.66	3.61 ± 1.30	4.14	< *0.001*
Lack of Relationship Skills	2.96 ± 1.75	3.15 ± 1.28	−1.56	0.121

**Table 6:** Differences in Relationship Sabotage by long-term family residence(*n* = 568)

Variable	Urban(*n* = 316)	Rural(*n* = 252)	*t*	*P*
Relationship Sabotage	3.29 ± 0.94	3.34 ± 0.89	−0.68	0.494
Defensiveness	3.20 ± 1.56	3.20 ± 1.48	0.02	0.985
Trust Difficulty	3.88 ± 1.52	3.76 ± 1.43	0.93	0.352
Lack of Relationship Skills	2.96 ± 1.50	3.21 ± 1.50	−1.97	0.049

**Table 7:** Differences in Relationship Sabotage by Left-Behind Experience(*n* = 568)

Variable	Yes(*n* = 168)	No(*n* = 400)	*t*	*P*
Relationship Sabotage	3.43 ± 0.88	3.27 ± 0.93	1.88	0.061
Defensiveness	3.33 ± 1.56	3.15 ± 1.50	1.24	0.214
Trust Difficulty	3.94 ± 1.51	3.79 ± 1.47	1.17	0.243
Lack of Relationship Skills	3.17 ± 1.52	3.03 ± 1.49	0.99	0.318

**Table 8:** Differences in Relationship Sabotage by Only-Child Status(*n* = 568)

Variable	Yes(*n* = 111)	No(*n* = 457)	*t*	*P*
Relationship Sabotage	3.18 ± 0.95	3.35 ± 0.91	−1.76	0.078
Defensiveness	3.00 ± 1.62	3.25 ± 1.50	−1.57	0.117
Trust Difficulty	3.76 ± 1.45	3.84 ± 1.49	−0.55	0.583
Lack of Relationship Skills	2.96 ± 1.60	3.10 ± 1.48	−0.84	0.399

**Table 9:** Differences in Relationship Sabotage by Migration Experience(*n* = 568)

Variable	Yes(*n* = 130)	No(*n* = 400)	*t*	*P*
Relationship Sabotage	3.34 ± 0.93	3.31 ± 0.92	0.44	0.659
Defensiveness	3.31 ± 1.60	3.17 ± 1.51	0.91	0.363
Trust Difficulty	3.82 ± 1.44	3.83 ± 1.50	−0.08	0.937
Lack of Relationship Skills	3.03 ± 1.39	3.08 ± 1.53	−0.29	0.776

### Reliability analysis

The Chinese version of the Relationship Sabotage Scale (RSS) demonstrated acceptable to good internal consistency. The overall Cronbach’s *α* was 0.792, with subscale *α* coefficients of 0.942 for Defensiveness, 0.832 for Trust Difficulty, and 0.949 for Lack of Relationship Skills, indicating acceptable reliability at both the total scale and subscale levels.

In addition, McDonald’s *ω* was calculated to provide a more robust estimate of reliability. As shown in Table 10, the overall scale *ω* = 0.909 (95% CI 0.889–0.924), and the average inter-item correlation was 0.447 (95% CI 0.396–0.495), indicating moderate correlations among items, consistent with a multidimensional scale structure.

**Table 10:** Overall Scale Reliability Statistics for the Relationship Sabotage Scale (*n* = 568)

Coefficient	Estimate	Std. Error	95% CI Lower	95% CI Upper
Coefficient ω	0.909	0.009	0.889	0.924
Average interitem correlation	0.447	0.025	0.396	0.495

To further examine item-level reliability, corrected item–total correlations (item-rest correlations) and ω if each item were deleted were computed (Table 11). Corrected item–total correlations ranged from 0.475 to 0.723 for most items, with lower values for Q5, Q9–Q12, which correspond to different subdimensions of the scale. Deleting any single item resulted in minimal changes in *ω* (0.896–0.910), indicating that all items contribute meaningfully to the scale’s overall reliability and that no items are redundant.

**Table 11:** Corrected Item–Total Correlations and Individual Item Reliability Statistics for the Relationship Sabotage Scale (*n* = 568)

Item	Coefficient ω (if item dropped)			Item-rest correlation		
	Estimate	95% CI Lower	95% CI Upper	Estimate	95% CI Lower	95% CI Upper
Q1	0.897	0.873	0.913	0.722	0.666	0.763
Q2	0.899	0.877	0.915	0.687	0.628	0.732
Q3	0.899	0.876	0.915	0.682	0.627	0.728
Q4	0.897	0.874	0.914	0.717	0.669	0.755
Q5	0.903	0.882	0.918	0.595	0.531	0.651
Q6	0.897	0.874	0.915	0.702	0.648	0.744
Q7	0.896	0.87	0.914	0.723	0.68	0.761
Q8	0.899	0.874	0.916	0.683	0.63	0.732
Q9	0.906	0.887	0.921	0.574	0.503	0.632
Q10	0.908	0.89	0.923	0.528	0.451	0.594
Q11	0.908	0.89	0.923	0.533	0.456	0.593
Q12	0.91	0.892	0.924	0.475	0.395	0.551

Overall, these analyses suggest that the Chinese RSS has satisfactory internal consistency, and all items can be retained. The inclusion of both overall reliability (Table 10) and item-level statistics (Table 11) ensures transparency and provides additional evidence of the scale’s psychometric properties.

### Measurement invariance

Using Sample 2, measurement invariance of the revised Chinese Relationship Sabotage Scale (RSS) was examined across five grouping variables: gender, only-child status, migration experience, left-behind experience, and long-term family residence. Multi-group confirmatory factor analysis (CFA) within a structural equation modeling framework was employed to sequentially test configural, metric, and scalar invariance.

The configural invariance model tested whether the factor structure (number of factors and pattern of item-factor loadings) was consistent across groups, without imposing equality constraints. Across all grouping variables, the configural models demonstrated good fit, confirming that the underlying factor structure is similar across groups.

The metric invariance model further constrained factor loadings to be equal across groups, allowing us to assess whether participants interpret the latent constructs similarly. Changes in model fit indices (*ΔCFI*, *ΔRMSEA*, *ΔSRMR*) relative to the configural model were compared against recommended thresholds (Chen & F.F, [9]: *ΔCFI* ≤ 0.010, *ΔRMSEA* ≤ 0.015, *ΔSRMR* ≤ 0.030 for configural → metric). Across all five grouping variables, *ΔCFI* ranged from −0.006 to 0.001, *ΔRMSEA* from −0.009 to 0.007, and *ΔSRMR* from 0.001 to 0.009, all within the acceptable ranges, supporting metric invariance.

The scalar invariance model additionally constrained item intercepts to be equal across groups, enabling valid comparisons of latent means. The fit changes from the metric model were also within thresholds (Chen & F.F, [9]: *ΔCFI* ≤ 0.010, *ΔRMSEA* ≤ 0.015, *ΔSRMR* ≤ 0.010 for metric → scalar), with maximum Δ*CFI* = 0.006, *ΔRMSEA* = 0.008, and *ΔSRMR* = 0.008 across all groups. These results support scalar invariance, suggesting that the RSS can be used to compare latent variable means across demographic groups.

Table 12 presents detailed model fit statistics for each step, including *χ*^*2*^, *df*, *CFI*, *RMSEA*, *SRMR*, and the changes in fit indices (*Δχ*^*2*^, *Δdf, ΔCFI*, *ΔRMSEA*, *ΔSRMR*). The explicit reporting of these indices and the use of established invariance thresholds ensure transparency and reproducibility of the measurement invariance analyses.

**Table 12:** Measurement Invariance of the Relationship Sabotage Scale Across Demographic Groups (*n* = 568)

Model	*X*^*2*^	*df*	CFI	RMSEA	SRMR	Δ*X*^*2*^	Δ*df*	ΔCFI	ΔRMSEA	ΔSRMR
Gender(Male = 240, Female = 328)										
Configural	232.711	122	0.929	0.044	0.041	NA	NA	NA	NA	NA
Metric	238.674	134	0.923	0.048	0.046	5.963	12	−0.006	0.004	0.005
Scalar	245.623	148	0.919	0.051	0.052	6.949	14	−0.004	0.003	0.006
Only child(Yes = 111, No = 457)										
Configural	228.184	122	0.932	0.043	0.041	NA	NA	NA	NA	NA
Metric	236.261	134	0.931	0.048	0.044	8.077	12	−0.001	0.005	0.003
Scalar	245.114	148	0.926	0.051	0.052	8.853	14	−0.004	0.003	0.008
Migration experience(Yes = 130, No = 438)										
Configural	401.423	122	0.925	0.043	0.041	NA	NA	NA	NA	NA
Metric	405.155	134	0.924	0.048	0.044	3.732	12	−0.001	0.005	0.003
Scalar	407.176	148	0.919	0.051	0.050	2.021	14	−0.005	0.003	0.006
Left-behind experience(Yes = 168, No = 400)										
Configural	292.861	122	0.923	0.039	0.038	NA	NA	NA	NA	NA
Metric	300.156	134	0.917	0.046	0.047	7.295	12	−0.006	0.007	0.009
Scalar	306.719	148	0.912	0.052	0.053	6.563	14	−0.005	0.006	0.006
long-term family residence(Urban = 316, Rural = 252)										
Configural	294.961	122	0.931	0.047	0.052	NA	NA	NA	NA	NA
Metric	303.218	134	0.932	0.038	0.053	8.257	12	0.001	−0.009	0.001
Scalar	310.775	148	0.926	0.046	0.055	7.557	14	−0.006	0.008	0.002

## Discussion

This study first verified the psychometric properties of the Relationship Sabotage Scale (RSS) in a Chinese university student population. The results showed that the Chinese revised version of RSS generally supports a three-factor structure(defensiveness, trust difficulty, lack of relationship skills), acceptable internal consistency, as well as evidence of convergent, discriminant, and known-groups validity. Additionally, it established configural invariance, metric invariance, and scalar invariance across gender, only child status, mobility experience, left-behind experience, and family residence. The Chinese version of RSS serves as an useful tool for assessing self-sabotage behaviors in intimate relationships among Chinese university students, providing preliminary support for its use as a measurement tool for research on intimate relationships. Its potential applied value in intervention contexts requires further clinical validation..

This study employed confirmatory factor analysis (CFA) to verify the three-factor structure of the Chinese revised version of RSS, consistent with findings from Peel et al. [38], Maden et al. [32], and Sadeghi et al. [44] in Australian, Turkish, and Iranian populations. Following cultural adaptation adjustments (e.g., reassigning item 5 from the trust difficulty dimension to the defensiveness dimension), the factor loadings of the Chinese version of RSS ranged from 0.562 to 0.899, with satisfactory model fit indices (*χ*^*2*^*/df* = 3.81, *RMSEA* = 0.070, *CFI* = 0.951, *TLI* = 0.930). Although overall model fit was acceptable in Sample 2, fit indices in Sample 1 were marginal (χ^2^/df = 5.64, RMSEA = 0.092, SRMR = 0.108, CFI = 0.899, TLI = 0.870), possibly reflecting sample-specific variability or multidimensionality of the construct. This limitation should be considered when interpreting the results. Internal consistency analysis revealed that the Cronbach’s *α* coefficient for the RSS scale was 0.792, with subscale coefficients (defensiveness, trust difficulty, lack of relationship skills) of 0.942, 0.832, and 0.949, respectively, indicating acceptable to good reliability. Item-level analyses indicated variability in corrected item–total correlations, which is consistent with the multidimensional structure of the scale. These findings suggest that the Chinese version of RSS demonstrates generally acceptable structural validity and reliability in the Chinese university student population and may be suitable for assessing self-sabotage behaviors in intimate relationships.

Convergent validity analysis indicated that each dimension of RSS was significantly correlated with the attachment avoidance and attachment anxiety dimensions of the Experiences in Close Relationships (ECR) scale (*r* = 0.097–0.716, *p* < 0.05), aligning with attachment theory expectations [8, 47]. The strong correlation between the trust difficulty dimension and attachment anxiety (*r* = 0.716, *p* < 0.001) reflects the heightened sensitivity to trust among individuals with insecure attachment in intimate relationships [31]. Discriminant validity analysis showed that RSS dimensions were negatively correlated with intimate relationship satisfaction and self-esteem (*r* = −0.507 to −0.226, *p* < 0.001), except for the non-significant correlation between trust difficulty and intimate relationship satisfaction. In Western cultural contexts, trust difficulty often reflects individual internal anxiety rather than direct relationship evaluation [39]. In contrast, in Chinese cultural contexts, intimate relationship satisfaction may depend more on external manifestations of the relationship, such as family support, emotional flow, intergenerational communication, and responsibility allocation [18, 24], rather than individual internal anxiety, which may explain the non-significant correlation. These results suggest that the Chinese version of RSS is capable of distinguishing self-sabotage behaviors from related psychological constructs, providing theoretical support for its application in intimate relationship research.

Criterion validity analysis revealed significant differences in self-sabotage behaviors by gender (*t* = 4.31, *p* < 0.001) and family residence (*t* = −1.97, *p* = 0.049), which may reflect the influence of gender roles and urban–rural differences on intimate relationship behaviors in the Chinese cultural context [53]. Influenced by Confucian thought, traditional Chinese culture emphasizes a gender role division of "men managing external affairs, women managing internal affairs," alongside social expectations of male dominance and female submissiveness [27]. When confronted with threats in intimate relationships (e.g., jealousy or conflict), males may be more likely to engage in self-sabotage behaviors, such as avoiding emotional expression or emphasizing external work pressures [57], to protect self-esteem or avoid emotional setbacks. This may partially explain why male university students scored higher than their female counterparts in self-sabotage behaviors in intimate relationships. Additionally, urban–rural differences reflect variations in socioeconomic status, cultural values, and relationship patterns [26], which may influence self-sabotage behaviors. Rural areas retain more traditional values, emphasizing family obligations and relationship stability [16], whereas urban areas, with better socioeconomic status, education levels, and cultural values [26], may foster stronger communication skills and greater opportunities for learning and practice, resulting in rural university students exhibiting weaker relationship skills compared to their urban counterparts.

Multi-group CFA results demonstrated that the Chinese version of RSS achieved configural, metric, and scalar invariance across variables such as gender, only child status, mobility experience, left-behind experience, and family residence [1, 42]. This findings suggest that the measurement properties of the Chinese version of RSS are equivalent across groups defined by gender (male, female), only child status (yes, no), mobility experience (yes, no), left-behind experience (yes, no), and family residence (urban, rural), consistent with findings by Maden et al. [32]. This cross-group consistency provides a basis for comparing levels of self-sabotage in intimate relationships across diverse groups.

The adaptation process for the RSS considered the collectivist characteristics of Chinese culture [53], family and bloodline relationships, and the concepts of "renqing" (human feelings) and "mianzi" (face). Notably, item 5 ("I often feel jealous of my partner") was reassigned to the defensiveness dimension. Chinese culture emphasizes a distinction between "in-groups" (e.g., partners, family) and "out-groups," which may lead to jealousy being expressed more as a defensive response against external threats (Miao, [36] rather than a lack of trust in the partner. Individuals may worry about their partner being attracted to "outsiders," exhibiting defensive behaviors such as monitoring or distancing rather than directly questioning their partner’s loyalty [53]. The translation and back-translation process ensured semantic equivalence, with item wording adjusted to align with the language habits and cultural background of Chinese university students. Interview assessments confirmed that the Chinese version of RSS has high clarity in its instructions and items, with strong cultural fit.

Importantly, the primary contribution of the present study lies in psychometric validation rather than clinical validation. Given the non-clinical, cross-sectional college samples, the findings should not be interpreted as supporting immediate clinical screening or diagnostic use. Further research involving clinical populations, longitudinal designs, and predictive validity analyses is necessary before establishing clinical applicability.

### Limitations

Despite providing evidence supporting the psychometric properties of the Chinese version of the RSS, several limitations should be acknowledged. First, Samples 1 and 2 were drawn from four universities in Jiangsu Province, with a relatively narrow age range (*M* = 18.82–19.18, *SD* = 1.51–1.65). Although these institutions represent northern, central, and southern areas of Jiangsu—with increasing levels of economic development from north to south—the geographic scope remains limited. This regional concentration and age homogeneity may restrict the generalizability of the findings to other age groups, non-student populations, or economically less developed regions. Future research should recruit participants from more diverse regions and educational or occupational backgrounds to enhance the external validity of the RSS across broader Chinese-speaking contexts. In addition, the marginal model fit observed in Sample 1 suggests that the factor structure should be interpreted with caution.

This study did not include clinical samples (e.g., individuals with anxiety or depression) and did not establish diagnostic validity or cutoff scores for distinguishing levels of self-sabotage. Given that self-sabotage behaviors may be misinterpreted as anxiety or depressive symptoms [38], future research should examine its applicability in clinical populations and evaluate its longitudinal stability and predictive validity before any diagnostic-related conclusions can be drawn.

Due to the cross-sectional design, test–retest reliability was not assessed, limiting conclusions regarding the temporal stability of the Chinese version of the RSS. Longitudinal studies are needed to evaluate test–retest reliability and ensure the scale’s stability over time.

Although measurement invariance was supported across five grouping variables within the present samples, the model involved a theoretically justified reassignment of Item 5. While residual correlations were not introduced, this adjustment may influence the replicability of the factor structure in independent samples. Furthermore, cross-cultural measurement invariance was not examined. Considering the theoretical differences between Chinese collectivistic norms and Western individualistic orientations, future studies should compare the measurement properties of the RSS across cultural contexts (e.g., Australia, Turkey, and Iran) and conduct independent sample validation to further assess the structural stability and generalizability of the scale.

## Conclusion

This study validated the Chinese version of the Relationship Sabotage Scale (RSS) among Chinese college students. The results provide preliminary support for the scale’s factor structure and internal consistency. Measurement invariance was generally supported across key demographic groups. These findings suggest that the scale may be a useful tool for assessing self-sabotaging behaviors in this population.

The Chinese RSS can help researchers better understand relationship sabotage in Chinese cultural contexts. It also provides a tool for future cross-cultural studies. Practically, it may inform interventions targeting young adults’ romantic relationships. However, some limitations should be noted. The samples were drawn from a limited geographic region and age range. No clinical populations were included. The factor structure involved a theoretically justified item reassignment and has not yet been cross-validated. Future studies should use independent and more diverse samples, examine longitudinal stability, and test cross-cultural generalizability. Overall, the Chinese RSS offers a potentially useful measurement tool. It contributes to research on self-sabotaging behaviors and highlights directions for future work.

## Supplementary Information

Supplementary Material 1.

## Data Availability

The data that support the findings of this study are available on request from the corresponding author.

## Appendix

**Table 13:** Correspondence Table of Items and Dimensions between the Original Scale and the Revised Scale

Item	Original Version English	Original Dimension	Revised Version Chinese	Revision Dimension
Q1	I constantly feel criticised by my partner	Defensiveness	我经常被我的伴侣批评	Defensiveness
Q2	My partner makes me feel a lesser person	Defensiveness	我的伴侣让我觉得我低人一等	Defensiveness
Q3	I get blamed unfairly for issues in my relationship	Defensiveness	在亲密关系中我受到了不公平的指责	Defensiveness
Q4	I often feel misunderstood by my partner	Defensiveness	我经常感到被伴侣误解	Defensiveness
Q5	I often get jealous of my partner	Trust Difficulty	我经常嫉妒我的伴侣	Defensiveness
Q6	I get upset about how much time my partner spends with their friends	Trust Difficulty	伴侣花费太多时间和朋友待在一起让我感到不安	Trust Difficulty
Q7	I believe that to keep my partner safe I need to know where my partner is	Trust Difficulty	我认为, 为了保证伴侣的安全, 我需要知道我的伴侣在哪儿	Trust Difficulty
Q8	I sometimes check my partner's social media profiles	Trust Difficulty	我有时会查看伴侣的社交媒体资料	Trust Difficulty
Q9	I am open to my partner telling me about things I should do to improve our relationship	Lack of Relationship Skills	我很乐意我的伴侣告诉我, 我可以做些什么来提升我们的亲密关系	Lack of Relationship Skills
Q10	I am open to finding solutions and working out issues in the relationship	Lack of Relationship Skills	我很乐意寻找解决办法, 解决我们亲密关系中的问题	Lack of Relationship Skills
Q11	I will admit to my partner if I know I am wrong about something	Lack of Relationship Skills	如果我做错了事情, 我会向伴侣承认错误	Lack of Relationship Skills
Q12	When I notice that my partner is upset, I try to put myself in their shoes so I can understand where they are coming from	Lack of Relationship Skills	当我注意到伴侣心烦意乱时, 我会站在伴侣的角度上换位思考, 这样我就能理解伴侣的想法	Lack of Relationship Skills
